# Estimating the overall survival benefit of adjuvant chemo‐endocrine therapy in women over age 50 with pT1‐2N0 early stage breast cancer and 21‐gene recurrence score ≥26: A National Cancer Database analysis

**DOI:** 10.1002/cam4.6584

**Published:** 2023-09-28

**Authors:** Nickolas Stabellini, Lifen Cao, Christopher W. Towe, Amanda L. Amin, Alberto J. Montero

**Affiliations:** ^1^ Case Western Reserve University School of Medicine Cleveland Ohio USA; ^2^ Faculdade Israelita de Ciências da Saúde Albert Einstein Hospital Israelita Albert Einstein São Paulo Brazil; ^3^ Division of Hematology and Oncology, Department of Medicine University Hospitals/Seidman Cancer Center, Case Western Reserve University School of Medicine Ohio Cleveland USA; ^4^ Division of Thoracic and Esophageal Surgery, Department of Surgery University Hospitals Research in Surgical Outcomes and Effectiveness (UH‐RISES), University Hospitals/Seidman Cancer Center, Case Western Reserve University School of Medicine Ohio Cleveland USA; ^5^ Division of Surgical Oncology, Department of Surgery University Hospitals Research in Surgical Outcomes and Effectiveness (UH‐RISES), University Hospitals/Seidman Cancer Center, Case Western Reserve University School of Medicine Ohio Cleveland USA

**Keywords:** adjuvant chemotherapy, adjuvant endocrine therapy, breast cancer, ER +, overall survival, recurrence score

## Abstract

**Background:**

Validation studies of the 21‐gene recurrence score (RS) previously demonstrated that adjuvant chemotherapy plus endocrine therapy (CET) was associated with a significant survival benefit in women with node negative breast cancer (BC) and RS >31. However, the TAILORx trial, did not quantify the benefit of adjuvant CET in older women with node negative hormone receptor positive (HR+) BC with RS ≥26. We hypothesized that CET would be associated with improved overall survival (OS) compared to endocrine therapy (ET) in women >50 with HR+/HER2‐node negative BC and RS ≥26.

**Methods:**

The National Cancer Database (NCDB) was queried to identify women >50 with RS ≥26 ER+/HER2‐BC pT1‐2N0M0. Chi‐square and logistic regression analysis determined the difference between ET and CET. OS was analyzed using a multivariable Cox model.

**Results:**

We included 16,745 women—4740 (28.3%) received ET and 12,005 (71.7%) received CET. Women who received CET had: moderately (OR = 1.853, *p* < 0.001) or poorly/undifferentiated tumors (OR = 3.875, *p* < 0.001), pT2 (OR = 1.356, *p* < 0.001), or lymph‐vascular invasion (OR = 1.206, *p* = 0.001). After accounting for demographic and oncologic factors, 5‐year OS rates were significantly superior in women receiving CET vs. ET alone (95.4% vs. 92.0%, Hazard Ratio = 0.680, *p* < 0.001).

**Conclusions:**

We observed that CET was associated with a clinically and statistically significant higher OS compared to ET alone in women >50 years of age with RS ≥26 pT1 and pT2 N0M0 HR+/HER2‐breast cancer, and which suggests that cytotoxic chemotherapy has an impact on reducing mortality that is independent of induction of premature ovarian failure.

## Graphical abstract

Chemotherapy plus endocrine therapy was associated with a clinically and statistically significant higher overall survival compared to endocrine therapy alone in women >50 years of age with RS >26 pT1 and pT2 N0M0 HR+/HER2‐breast cancer, suggesting that cytotoxic chemotherapy has an impact on reducing mortality that is independent of induction of premature ovarian failure.

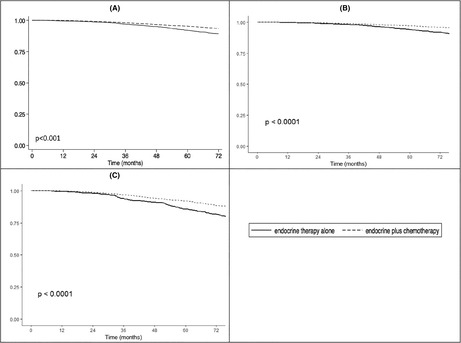

## 1 INTRODUCTION

Female breast cancer (BC) is the most commonly diagnosed cancer worldwide, with an estimated 2.3 million new cases in 2020, equating to more than one diagnosis every 18 s.^1^, ^2^ In the United States alone, approxiately 297,790 new BC cases are estimated in 2023, accounting for 31% of all cancer diagnoses among women,  and 43,170 estimated deaths to metastatic breast cancer.^3^, ^4^ The management of BC depends on key factors such as TNM staging, receptor status, and recurrence score, which collectively categorize it as early or advanced (metastatic) disease.^5^, ^6^, ^7^ For early‐stage BC, surgical intervention is typically the course of action for operable cases, and adjuvant therapy may be recommended in instances of heightened recurrence risk.^6^, ^8^

In recent years, discussions have centered on the potential benefits of augmenting adjuvant endocrine therapy (ET) with adjuvant chemotherapy for early‐stage BC.^8^, ^9^ Meta‐analyses of adjuvant chemotherapy trials have consistently demonstrated a significant overall survival (OS) benefit of adjuvant chemo plus endocrine therapy (CET) in women with hormone receptor positive (HR+) BC across different age groups, with a greater benefit observed for poly‐chemotherapy regimens that include taxanes and anthracyclines.^10^ These findings were further corroborated by retrospective studies.^11^ In the Early Breast Cancer Trialists' Collaborative Group meta‐analysis (EBCTCG), a proportionally greater benefit of adjuvant CET compared to tamoxifen alone in reducing 5‐year BC recurrence probabilities was observed for women under 50 years of age than those 50–64, with absolute 5‐year gains of 7.6% versus 4.9%, respectively.^10^ A recently published meta‐analysis by the EBCTCG showed that recurrence rates were 14% lower on average in breast cancer patients with adjuvant taxane regimens that included anthracyclines compared to those without. Adjuvant combination anthracycline and taxane chemotherapy regimens were beneficial even in women older than 50 years of age, irrespective of ER status.^12^

This differential benefit of adjuvant CET, in women under age 50, and therefore more likely to be pre‐menopausal, has been hypothesized to be due to chemotherapy‐induced ovarian function suppression (OFS) in this subgroup of patients.^13^ Although this hypothesis has not been directly tested in large randomized clinical trials, prior studies have demonstrated ovarian ablation plus goserelin to have a similar benefit as CMF chemotherapy in premenopausal women with node positive HR+ BC.^14^

The more recently conducted TAILORx and MINDACT trials, which evaluated the impact of two different genomic tests in identifying clinical high risk early stage HR+ BC patients who do not require adjuvant chemotherapy, both observed that the primary benefit of adjuvant CET was restricted primarily to premenopausal patients (<50 years).^7^, ^15^, ^16^ TAILORx for example, found a significant interaction between age and CET (*p* = 0.004), such that some benefit of chemotherapy was found in women ≤50 years of age with a mid‐range 21‐gene recurrence score (RS) of 16–25,^2^ and no benefit of adjuvant CET in women >50.^7^ Similarly, the RxPONDER trial demonstrated that adjuvant chemotherapy was not beneficial in post‐menopausal patients with HR+/HER2‐BC, 1–3 positive nodes, and RS <25.^17^ Tissue blocks from the NSABP B20 study, were utilized to retrospectively validate the RS.^18^ The study published by Paik et al. demonstrated that women with node negative BC with high RS (≥31) derived a significant benefit from CET, while women with low RS‐BC (<18) derived minimal, if any, benefit from chemotherapy.^19^

Neither the initial validation study by Paik et al. nor TAILORx were designed to estimate the magnitude of benefit of adjuvant CET in post‐menopausal women with node negative HR+ BC. We therefore performed a real world analysis utilizing National Cancer Database (NCDB) to estimate the overall impact of adjuvant CET compared to ET alone on OS in women age > 50 with HR+/HER2‐pT1‐2N0M0 BC and RS ≥26. We hypothesized that given the benefit of adjuvant CET regardless of age noted in EBCTCG meta‐analyses, CET would be associated with a statistically significant OS benefit in postmenopausal women >50 compared to ET alone.

## 2 METHODS

### 2.1 Data collection and data elements

A retrospective cohort study of the NCDB was performed.^20^ Jointly sponsored by the American College of Surgeons and the American Cancer Society, NCDB is a clinical oncology database sourced from hospital registry data representing more than 70% of newly diagnosed cancer cases nationwide.^20^ The database covers more than 1500 Commission on Cancer (CoC)‐accredited facilities.^20^ Definition of the database variables are available from the dictionary of NCDB Participant Use Data File (http://ncdbpuf.facs.org). The hospitals participating in the CoC NCDB are the source of the de‐identified data used herein; they have not verified and are not responsible for the statistical validity of the data analysis or the conclusions derived by the authors.

### 2.2 Patient cohort

The NCDB was queried to identify female patients age >50 with HR+/HER2‐pT1‐2N0M0 BC and RS ≥26 from 2004 to 2017. Clinical staging data for the cohort was based on TNM classification in American Joint Committee on Cancer (AJCC) 7th edition.^21^ Patients were excluded if they did not have breast surgery, did not take ET, or were missing critical study information (e.g., follow‐up data). The cohort was stratified by adjuvant therapy: ET alone or CET.

### 2.3 Statistical analysis

The primary outcome was OS. Analysis included univariable comparison of patient factors associated with CET (vs. ET). To compare the two groups, Wilcoxon rank‐sum test was utilized for continuous variables and chi‐square for categorical data. Difference is OS between the groups was analyzed using Kaplan–Meier survival estimates and compared through log‐rank test, with a subgroup analysis by T stage. To control for confounding effects, multivariable Cox proportional hazard analysis was performed.

All statistical analysis was performed using STATA/MP, version 16.0 (Stata Corp LLC). Institutional Review Board (IRB) approval was exempted by the University Hospitals Cleveland Medical Center IRB as all data is de‐identified.

## 3 RESULTS

**FIGURE 1:**
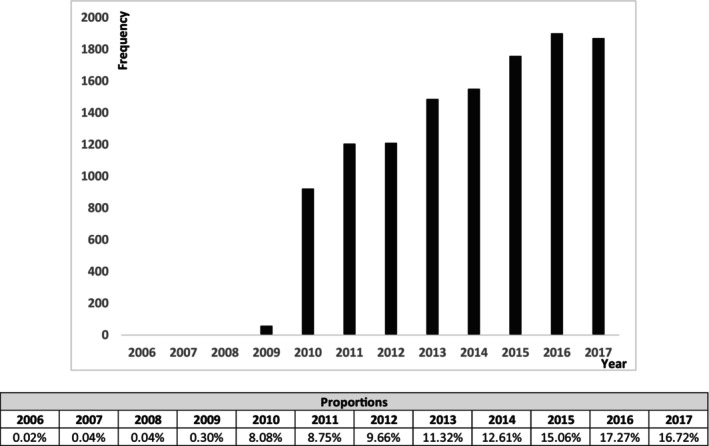
Chemotherapy utilization among women >50 with pT1‐2N0M0 HR+HER2‐breast cancer and RS ≥26. Data is shown as individual patient numbers by year.

**FIGURE 2:**
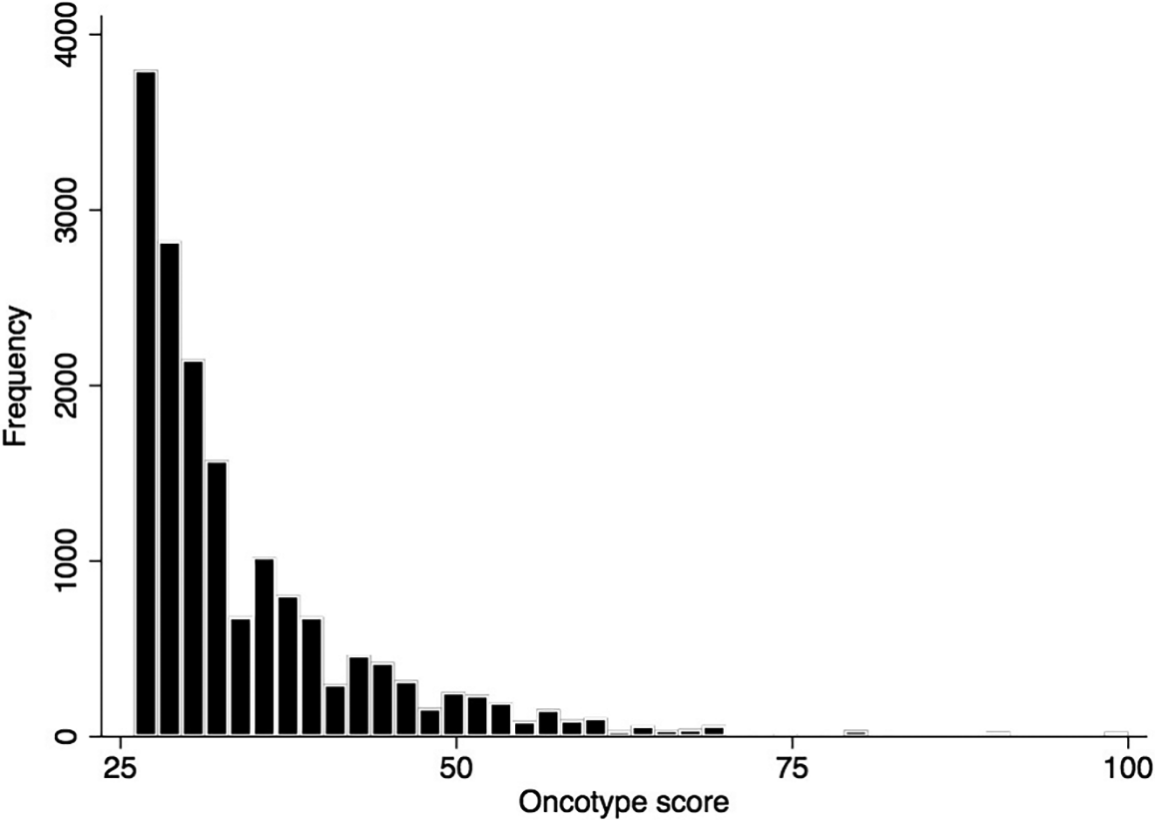
Frequency histogram of the 21‐gene RS ≥26 distribution among patients >50 years of age with pT1‐2N0M0 HR+HER2‐breast cancer.

A total of 16,745 BC patients met the inclusion criteria. Of these, 4740 (28.3%) received ET and 12,005 (71.7%) received CET. We observed that CET use in patients with pT1‐2N0 BC in women 50 and older increased over time, from 62 in 2009 to over 1,800 patients by 2017 (Figure 1). The histogram of RS among patients is shown in Figure 2, with most patients having a RS of 26–31 (52.39%). Patient characteristics stratified by adjuvant treatment approach is shown in Table 1. Adjuvant treatment did not significantly differ by race, facility area, distance to the hospital, or type of axillary surgery. However, patients who received CET were: younger (61 vs. 66 years, *p* < 0.001), had lower comorbidity scores (Charlson‐Deyo score of 0 in 83.2% vs. 80.3%, *p* < 0.001), higher tumor grade (poorly differentiated or undifferentiated 53.7% vs. 33.8%, *p* < 0.001), had lymphovascular invasion (16.2% vs. 12.3%, *p* < 0.001), and were more likely to have pT2 tumors (32.6% vs. 25.4%, *p* < 0.001). Moreover, patients who received CET were more likely to have private insurance (60.6% vs. 42.8%, *p* < 0.001), receive treatment at an academic/research facility (31.1% vs. 28.5%, *p* = 0.002), and have undergone mastectomy instead of breast conservation (23.3% vs. 20.8%, *p* < 0.001).

**TABLE 1:** Demographic, clinical and treatment characteristics of women over 50 years of age, pT1‐2N0M0 HR+HER2‐breast cancer, and 21‐gene RS ≥26:NCDB 2004–2017.

Characteristics	Endocrine therapy alone (*n* = 4,740)		Endocrine plus chemotherapy (*n* = 12,005)		
	NO.	%	NO.	%	*p* value
Age, years	66 (60–72)		61(56–67)		<0.001
Race					0.620
White	4,062	86.43	10,205	85.84	
Black	449	9.55	1,182	9.94	
Asian and other	189	4.02	501	4.21	
Charlson‐Deyo Score					<0.001
0	3,808	80.34	9,985	83.17	
1	704	14.85	1,614	13.44	
2	175	3.69	301	2.51	
3	53	1.12	105	0.87	
Insurance					<0.001
Public	2,636	56.31	4,564	38.4	
Private	2,004	42.81	7,198	60.56	
Not insured	41	0.88	124	1.04	
Facility Type					0.002
Community cancer program	346	7.3	801	6.67	
Comprehensive community cancer program	2,034	42.91	4,850	40.4	
Academic/research program	1,349	28.46	3,730	31.07	
Integrated network cancer program	1,011	21.33	2,624	21.86	
Facility Area					0.264
Metro	3,944	84.82	10,045	85.81	
Urban	629	13.53	1,478	12.63	
Rural	77	1.66	183	1.56	
Distance to the hospital (miles)					0.561
0.1/20	3,139	75.38	7,784	75.35	
20.1/40	579	13.9	1,467	14.2	
40.1/60	220	5.28	485	4.7	
60.1/max	225	5.4	590	5.71	
Grade					<0.001
Well differentiated	688	14.92	763	6.53	
Moderately differentiated	2,363	51.25	4,647	39.8	
Poorly differentiated	1,554	33.7	6,245	53.49	
Undifferentiated	6	0.13	21	0.18	
Lympho‐vascular invasion					<0.001
Not Present	3,702	87.7	8924	83.82	
Present	519	12.3	1,723	16.18	
Breast surgery type					<0.001
Partial mastectomy	3,755	79.22	9,210	76.72	
Unilateral mastectomy	720	15.19	1,790	14.91	
Bilateral mastectomy	265	5.59	1,005	8.37	
Axillary surgery type (after 2012)					0.43
SLNB	3,202	81.85	7,935	81.26	
ALND	695	17.77	1,778	18.21	
No axillary surgery	15	0.38	52	0.53	
pT Stage					<0.001
T1	3,533	74.53	8,088	66.70	
T2	1,207	25.46	3,917	32.62	

Abbreviations: ALND, axillary lymph node dissection; RS, recurrence score; SLNB, sentinel lymph node biopsy.

A multivariable logistic regression was performed to determine whether any clinical factors were independently associated with CET (Table 2). We found that increasing age (OR = 0.924, 95% CI 0.918–0.930, *p* < 0.001), African American race (OR = 0.859, 95% CI 0.753–0.980, *p* = 0.024), and high comorbidity score (OR = 0.763, 95% CI 0.632–0.921, *p* = 0.005) were associated with a significantly lower likelihood of receiving CET. Conversely, high tumor grade (moderately differentiated OR = 1.853, 95% CI 1.631–2.106, *p* < 0.001; poorly differentiated or undifferentiated OR = 3.875, 95% CI 3.398–4.419, *p* < 0.001), the presence of lympho‐vascular invasion (OR = 1.206, 95% CI 1.076–1.352, *p* < 0.001), and T2 stage (OR = 1.356, 95% CI 1.241–1.482, *p* < 0.001) were associated with a significantly higher probability of receiving CET.

**TABLE 2:** Multivariable logistic regressions for predictors of receipt of CET versus ET alone in women over 50 years with pT1‐2N0M0 HR+HER2‐breast cancer and 21‐gene RS ≥26: NCDB 2004–2017.

CET vs. ET	Odds ratio (OR)	95% Confidence interval		*p*‐value
>Age	0.924	0.918	0.930	<0.001
Race				
White	Reference			
African American	0.859	0.753	0.980	0.024
Asian or others	0.880	0.727	1.065	0.190
Charlson‐Deyo score				
0	Reference			
1	0.953	0.853	1.064	0.387
2+	0.763	0.632	0.921	0.005
Facility type				
Community	Reference			
Comprehensive	1.029	0.878	1.207	0.722
Academic	1.110	0.942	1.309	0.211
Integrated	1.095	0.925	1.297	0.293
Insurance status				
Public insurance	Reference			
Private insurance	1.065	0.966	1.174	0.207
Not insured	0.849	0.560	1.286	0.439
Grade				
Well differentiated	Reference			
Moderately differentiated	1.853	1.631	2.106	<0.001
Poorly or undifferentiated	3.875	3.398	4.419	<0.001
Lympho‐vascular invasion	1.206	1.076	1.352	0.001
T Stage				
T1	Reference			
T2	1.356	1.241	1.482	<0.001

**FIGURE 3:**
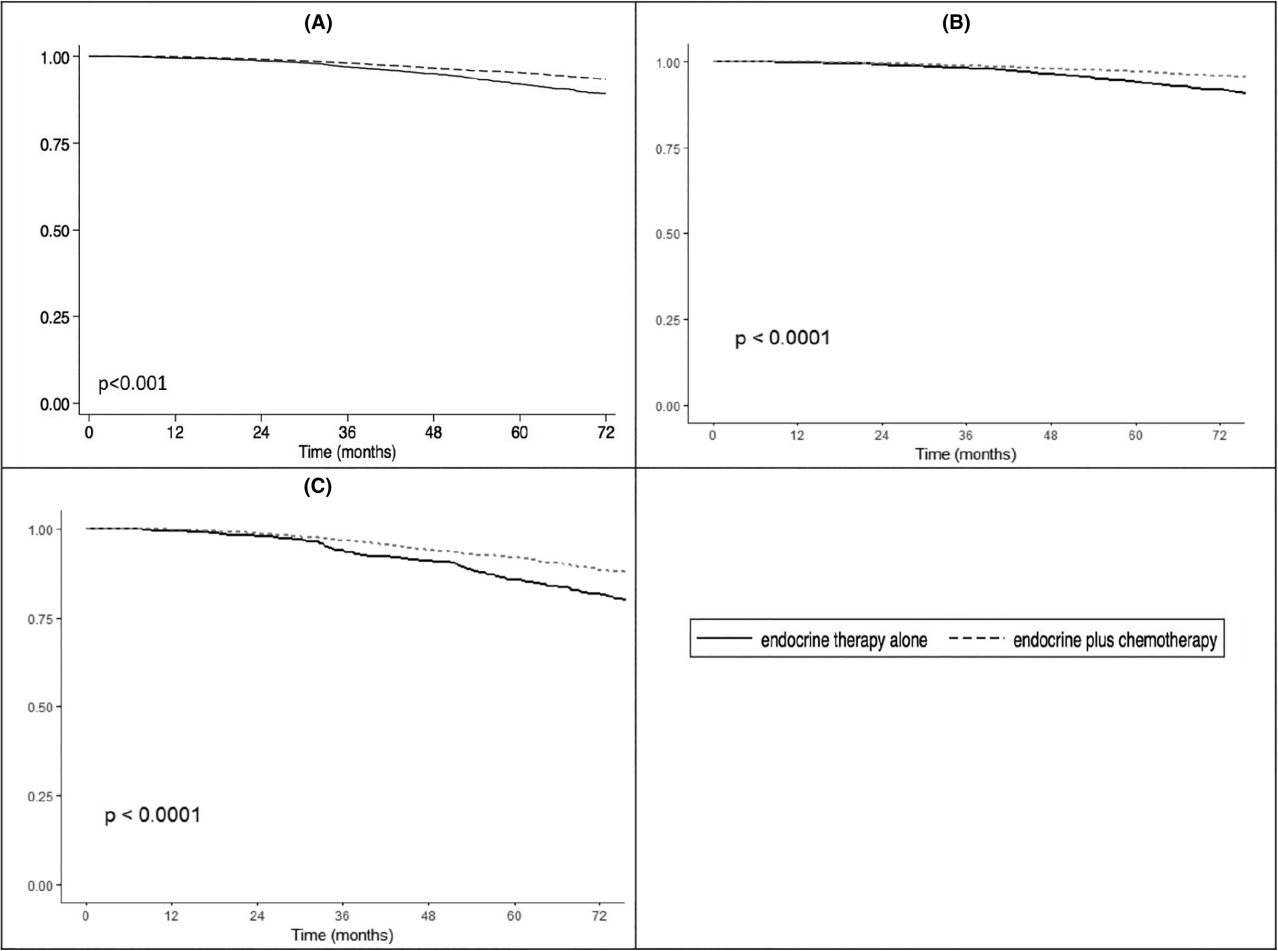
Kaplan–Meier analysis of overall survival of women over 50 with pT1‐2N0M0 HR+HER2‐breast cancer and RS ≥26, comparing CET vs. ET. Comparison of groups performed using log‐rank test. (A) Analysis of the entire cohort revealed that CET compared to ET alone was associated with significantly improved (*p* < 0.001) 3‐year (98.1% vs. 97.0%) and 5‐year OS rates (95.4% vs. 92.0%), respectively. (B) OS analysis of CET compared to ET alone (*p* < 0.001) in patients with pT1N0 disease. (C) OS of CET compared to ET alone (*p* < 0.001) in patients with pT2N0 disease.

Kaplan–Meier estimates demonstrated that among women over age 50 with pT1‐2N0M0 HR+HER2‐BC and RS ≥26, those who received adjuvant CET had a significantly higher OS compared to patients who received ET alone, independent of T stage (*p* < 0.001, Figure 3). Three and five‐year overall survival rates for CET versus ET were 98.1% versus 97.0%, and 95.4% versus 92.0%, respectively.

Multivariable Cox proportional hazard regression revealed that after controlling for demographic, clinical pathologic, age, comorbidities, and T stage, adjuvant CET was still associated with a significantly higher survival probability (HR = 0.680, 95% CI 0.586–0.790, *p* < 0.001, Table 3). Older age (HR = 1.029, 95% CI 1.018–1.041, *p* < 0.001), high comorbidity score (Charlson‐Deyo Score of 1 HR = 1.281, 95% CI 1.068–1.537, *p* < 0.008; Charlson‐Deyo Score ≥2 HR = 2.543, 95% CI 1.995–3.240, *p* < 0.001), higher grade tumor (moderately differentiated HR = 1.379, 95% CI 1.010–1.884, *p* = 0.043; poorly differentiated HR = 1.948, 95% CI 1.434–2.648, *p* < 0.001), the presence of lymphovascular invasion (HR = 1.639, 95% CI 1.393–1.928, *p* < 0.001), and pT2 stage (HR = 2.190, 95% CI 1.905–2.519, *p* < 0.001) were all associated with a significantly higher death risk.

**TABLE 3:** Cox proportional hazard regression for overall survival of women >50 with pT1‐2N0M0 HR+HER2‐breast cancer, and 21‐gene RS ≥26: NCDB 2004–2017.

	Hazard Ratio (HR)	95% Confidence interval		*p*‐value
CET vs. ET	0.680	0.586	0.790	<0.001
Age	1.029	1.018	1.041	<0.001
Race				
White	Reference			
African American	1.183	0.951	1.472	0.132
Asian or others	0.779	0.522	1.161	0.220
Charlson‐Deyo score				
0	Reference			
1	1.281	1.068	1.537	0.008
2+	2.543	1.995	3.240	<0.001
Facility type				
Community	Reference			
Comprehensive	0.730	0.570	0.934	0.012
Academic	0.632	0.486	0.821	0.001
Integrated	0.564	0.429	0.741	<0.001
Insurance status				
Public insurance	Reference			
Private insurance	0.662	0.556	0.790	<0.001
Not insured	0.477	0.177	1.283	0.142
Grade				
Well differentiated	Reference			
Moderately differentiated	1.379	1.010	1.884	0.043
Poorly differentiated	1.948	1.434	2.648	<0.001
Lympho‐vascular invasion	1.639	1.393	1.928	<0.001
T Stage				
T1	Reference			
T2	2.190	1.905	2.518	<0.001

## 4 DISCUSSION

To the best of our knowledge, this is the first study to estimate the overall survival benefit of adjuvant CET in women over age 50 with pT1‐2 N0 early stage breast cancer and 21‐gene recurrence score ≥ 26 using the NCDB, a nationally representative dataset from the United States. Analyzing data from 16,745 patients with BC, we demonstrated that CET was associated with 32% lower risk of death and higher 3‐ and 5‐year OS rates (98.1% vs. 97.0%, and 95.4% vs. 92.0%, respectively), when compared to ET alone in women >50 years of age with RS ≥26 pT1‐ pT2 N0M0 HR+/HER2‐BC.

In addition to our primary findings, we also found that the CET utilization rate was 71.7% within the investigated cohort. We found key differences between patients who received only adjuvant ET and those who received CET which included: younger age, lower comorbidity scores, higher tumor grade, lymphovascular invasion, pT2 tumors, a higher prevalence of private insurance coverage, a greater likelihood of receiving care at academic/research institutions, and higher liklihood of mastectomy. Furthermore, through the application of a regression framework, we found that that older age, African American ethnicity, and elevated comorbidity scores were significantly associated with a diminished likelihood of receiving CET. Conversely, higher tumor grade, lymphovascular invasion, and pT2 stage tumors were robustly linked to an higher probability of adjuvant CET.

Data from MINDACT, TAILORx, and RxPonder phase III trials—genomically driven adjuvant BC clinical studies—suggest that most of the benefit of adjuvant chemotherapy in reducing HR+/HER2 negative BC mortality in patients with genomically low risk tumors is confined to women under age 50 (and therefore more likely pre‐menopausal).^7^, ^15^, ^16^, ^17^ This observed benefit is likely an indirect consequence, rather than a direct antitumor effect, and is associated with cytotoxic chemotherapy triggering premature ovarian failure.^13^ However, these results are somewhat discordant from EBCTCG meta‐analyses of adjuvant chemotherapy trials, which have consistently shown a benefit of CET in women with HR+ breast cancer that is independent of age.^10^ Given that most women 50 and older with node‐negative pT1‐2N0 HR+/HER2‐BC are unlikely to benefit significantly from adjuvant chemotherapy alone, accurately identifying which node‐negative patients in this age group with HR+ breast cancer would benefit from adjuvant CET becomes crucial.^22^ Furthermore, estimating the relative impact of CET compared to ET in reducing breast cancer mortality can provide valuable guidance for informed decision‐making by both healthcare professionals and patients. Our study addresses these critical questions and reaffirms the advantages of CET, and align with the findings from the EBCTCG meta‐analyses.

The TAILORx study was specifically designed to assess the influence of adjuvant CET versus ET in women diagnosed with node‐negative, HR+ BC and a RS of 11–25.^7^ Notably, in this trial, all individuals with an RS exceeding 26 were exclusively assigned to receive CET. Consequently, the study does not provide insights into the comparative impact of adjuvant CET versus ET within this specific population. A recently published study by Sparano et al. attempted to address this question by performing a secondary analysis of data from the TAILORx cohort, which included 1,389 women with hormone receptor–positive, HER2‐negative, node‐negative BC, and RS of ≥26.^23^ In this cohort, where all patients were assigned to receive adjuvant CET, and approximately only 6% received ET alone.^23^ The estimated 5‐year OS was 96% (SE 0.6%), compared to 90.7% (SE 3.7%) for those who did not receive CET.^23^ These results are in line with our own 5‐year OS rates of 95.4% and 92% for CET and ET, respectively. However, it is important to note that the median age of these 1,389 women was 56 years, but the results of CET versus ET were not stratified by age, and the overall number of patients who received ET alone was very small (*n* = 89, 6%). Therefore, the benefit of CET over ET reported here involves (premenopausal) women under the age of 50, since 30% of analyzed patients were under 50.

From a clinical standpoint, the outcomes of our study contribute to the growing body of evidence endorsing the utilization of the 21‐gene RS in guiding decisions regarding adjuvant chemotherapy in women with HR+/HER2‐BC.^7^ Moreover, our findings underscore the relevance of considering CET as a viable adjuvant treatment option for postmenopausal women even in cases of early‐stage disease, given its promising implications for positively imacting OS. Additionally, tools such as PREDICT UK may prove valuable in estimating the potential benefits of various adjuvant strategies within this patient population.^24^, ^25^, ^26^

Our study does have several limitations. The NCDB does not include local regional recurrence and disease‐free survival, thus our analysis of long‐term outcomes is limited to OS. Similarly, we do not have access to data regarding to the intensity of ER/PR expression, which is not included in NCDB. Another disadvantage of NCDB is that is does not include specific information on the type of adjuvant chemotherapy administered (anthracycline vs. non‐anthracycline) or ET (aromatase inhibitor vs. tamoxifen), nor the overall duration of ET—granular information, which we know from the published literature significantly influences both recurrence‐free and OS. Another limitation in our study is that age over 50 is an imperfect surrogate for menopausal status, but there were no other alternatives since NCDB does not capture menopausal status. Finally, the NCDB only receives data from Commission on Cancer (CoC) accredited hospitals, and therefore excludes patients treated in many non‐CoC accredited centers in the United States. On the other hand, the strengths of our study lie in the fact that it addresses a crucial clinical question that remains unaddressed by randomized clinical trials. Considering the improbability of any future clinical trial providing an answer to this question, the utilization of real‐world data is a reasonable approach. Additionally, our study benefits from the overall quality and substantial sample size of the real‐world data we have employed.

In summary, using age as a surrogate for menopausal status, adjuvant CET does have a clinically and statistically significant impact in improving OS in women over 50 with pT1‐2 HR+/HER2‐, node negative BC and RS ≥26. Based on a real world analysis using NCDB, the overall impact of CET over ET in this patient population was a 32% reduction of death risk, translating into a 5‐year OS difference of approximately 2%. These findings are consistent with data from the EBCTCG, which have demonstrated an OS benefit of adjuvant CET in women over 50 with node negative HR+ BC. Future studies should prioritize prospective designs, delve into diverse patient populations, evaluate the impact of CET in various subgroups, and thoroughly assess the feasibility and effectiveness of integrating CET strategies within different healthcare systems.

## AUTHOR CONTRIBUTIONS

**Nickolas Stabellini:** Conceptualization (equal); methodology (equal); writing – original draft (equal); writing – review and editing (equal). **Lifen Cao:** Conceptualization (equal); data curation (equal); formal analysis (lead); methodology (equal); writing – original draft (lead); writing – review and editing (equal). **Christopher W Towe:** Conceptualization (equal); methodology (equal); writing – original draft (equal); writing – review and editing (equal). **Amanda L Amin:** Methodology (equal); writing – original draft (equal); writing – review and editing (equal). **Alberto J Montero:** Conceptualization (equal); methodology (equal); project administration (lead); resources (equal); supervision (lead); validation (equal); writing – original draft (equal); writing – review and editing (equal).

## FUNDING INFORMATION

NS is supported through funding from the Sociedade Beneficente Israelita Brasileira Albert Einstein on the program “Marcos Lottenberg & Marcos Wolosker International Fellowship for Physicians Scientist–Case Western”.

## ETHICS STATEMENT

This study was provided Institutional Review Board (IRB) exemption due to the de‐identified data source.

## MEETING PRESENTATION

The abstract of this study was presented as a poster presentation at the American Society of Clinical Oncology Annual Meeting June 3–7, 2022, Chicago, IL.

## Data Availability

NCDB data is available through an application process to investigators associated with CoC‐accredited cancer programs.
